# Acoustic differentiation and behavioral response reveals cryptic species within *Buergeria* treefrogs (Anura, Rhacophoridae) from Taiwan

**DOI:** 10.1371/journal.pone.0184005

**Published:** 2017-09-06

**Authors:** Ying-Han Wang, Yu-Wei Hsiao, Ko-Huan Lee, Hui-Yun Tseng, Yen-Po Lin, Shohei Komaki, Si-Min Lin

**Affiliations:** 1 Department of Life Science, National Taiwan Normal University, Taipei, Taiwan; 2 Institute of Ecology and Evolutionary Biology, National Taiwan University, Taipei, Taiwan; 3 Department of Biology, National Museum of Natural Science, Taichung, Taiwan; 4 Division of Zoology, Taiwan Endemic Species Research Institute, Nantou, Taiwan; 5 Division of Developmental Science, Graduate School for International Development and Cooperation, Hiroshima University, Hiroshima, Japan; 6 Global Career Design Center, Hiroshima University, Hiroshima, Japan; University of Sao Paulo, BRAZIL

## Abstract

*Buergeria japonica* is a widely distributed treefrog occurring from Ryukyu Archipelago to Taiwan. Across this wide distributional range, we combined molecular, acoustic, morphological, and behavioral characters to clarify the taxonomic status among these insular populations. Genetic differentiation in mitochondrial sequences indicated an over 16% divergence among two deeply divergent clades: Japanese clade distributes in Ryukyu Archipelago and northwestern drainages of Taiwan, while Taiwanese clade distributes in the remaining drainages on Taiwan. The Taiwanese clade can be distinguished from the nominative species not only by molecular and morphological differences, but also distinguishable by considerable acoustic differentiation, which is extraordinarily noticeable for an additional type of long call that never recorded from Japanese clade. The two clades form a parapatric distribution pattern with narrow contact zones both in western and eastern Taiwan. Playback experiments indicated that male frogs show significantly stronger defensiveness against conspecific calls rather than heterospecific calls, indicating that these signals play a crucial role in species recognition. Here we describe the Taiwanese clade as a new species; the behavioral response and the magnitude of gene flow across their contact zones are especially worth for detailed studies.

## Introduction

The rapid development of molecular techniques has improved our ability to uncover cryptic species and thus provided evidences on uncovered biodiversity [1, 2]. However, delimitating species based solely on molecular evidence is insufficient in cryptic species diagnosis [3, 4, 5]. Acoustic signal, as a critical factor in species recognition and mate choice, is a character that should be included as diagnostic criteria for anurans. Frog calls play the role as a diagnostic signal that supports intraspecific recognition or reproductive isolation [6, 7, 8]; while acoustic signals have been shown to be vital to mate choice and resource defense in anurans [8, 9].

The old world tree frogs, family Rhacophoridae, comprises roughly 400 species from 18 genera [10, 11]. The basal group of Rhacophoridae is a monophyletic subfamily, Buergeriinae, which comprises only one genus and four species [12, 13] distributed exclusively on East Asian islands: *Buergeria buergeri* (Temminck & Schlegel 1838) in southern Japan; *B*. *oxycephala* (Boulenger 1900) in Hainan Island, southern China; *B*. *robusta* (Boulenger 1909) endemic to Taiwan Island and *B*. *japonica* (Hallowell 1861) recorded both from Taiwan and the Ryukyu Archipelago. Compared to other rhacophorid members, this genus is characterized by flattened body shape and comparatively strong hind limbs, which might represent their adaptation to fast-flowing mountain streams. With high dependency to this specific habitat, population differentiation of *Buergeria* frogs shows strong association to drainage basins and makes them a good model for phylogeographic studies [14].

The Japanese stream treefrog (also known as Ryukyu kajika frog), *Buergeria japonica*, widely distributed on the East Asian Arc from Ryukyu Archipelago to Taiwan, is the only species among the four which shows a wide geographic distribution across different island groups. In this region, repetitive glacial cycles contributed to the diversification and distribution of terrestrial vertebrates, and led to the extremely high endemism of herptile fauna [15, 16, 17]. Compared to those restrictedly distributed species, the wide-spread distribution of *B*. *japonica* was explained by their unusual high tolerance to salty water [18]; they represent one of the very few cases of amphibians which might be capable of accidental over-seas dispersal [19].

On the other hand, within-island topology also contributed to differentiation among amphibian populations in this region. Deep intraspecific genetic divergences have been reported from several amphibians on Taiwan [20, 21]. In an extreme case, the genetic landscape of a congener (*Buergeria robusta*) represented a perfect congruence to the topography on this island [14]. Similar situation was discovered from *Buergeria japonica* in several recent studies [19, 22]; all of which clustered populations in northwestern Taiwan and Ryukyus in the same clade (hereafter defined as the “Japanese clade”), while populations from the eastern and southern Taiwan formed another (defined as the “Taiwanese clade”). The reported deep molecular divergence indicated the necessity to re-evaluate the taxonomic status of *B*. *japonica* across different regions, while behavioral traits are critical to solve this debate.

In this study, we evaluated the taxonomic status of Japanese stream treefrog by a combination of molecular, acoustic, morphological, and behavioral characters. Our results indicated prominent acoustic differences which correspond with their genetic differentiation. We further demonstrate that male frogs are able to distinguish conspecific versus heterospecific calls in a parapatric contact zone. We thus describe the eastern and southern populations of *Buergeria* in Taiwan as a new species, which represents a parapatric distribution with respect to *B*. *japonica sensu stricto*.

## Materials and methods

### Molecular sample collections and sequencing

**Fig 1:**
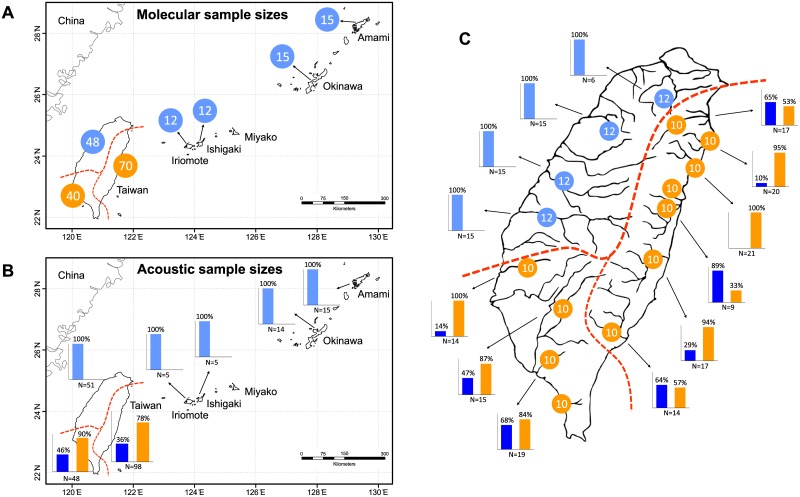
Sampling regimes of *Buergeria japonica sensu lato* in this study. (A) Sample localities, sample sizes, and genetic assignments from molecular evidence; (B) sample localities, sample sizes, and call types from acoustic evidence; and (C) the fine-scale sampling regime in Taiwan. Populations from Ryukyu and northwestern Taiwan (Japanese clade) could express only one type of long calls (Type 1A in light blue), but those from eastern and southern Taiwan (Taiwanese clade) are capable to express two: one very similar to the former (Type 1B in dark blue), and the other exclusively occurring in this region (Type 2 in orange). Arabic numerals in the pie charts indicate samples sizes for molecular analysis; while percentages in column charts indicate the population ratio in each drainage which was recorded to express either types of the calls.

A total of 212 specimens were collected from 19 drainages across 7 geographic regions during 2008–2010 (Fig 1; Table 1). Sampling of this study did not contain protected areas nor protected species, and was certificated under the rules of Wildlife Conservation Acts. The fourth toe of the hind limb was collected and stored in 95% ethanol, and the frog was released immediately back to their original habitats. Total genomic DNA was extracted by using DNeasy Blood & Tissue Kit (Qiagen, Valencia, CA) according to the manufacturer’s protocols. We suspended DNA in 1X TE buffer and stored at -20°C.

**Table 1:** Region, locality, abbreviation, sample sizes (acoustic, morphological, molecular, and number of mitochondrial haplotypes), and GenBank accession numbers for each *Buergeria japonica* population used in this study.

Species	Region	Sample locality	Abbr.	Sample sizes				GenBank accession numbers
				Acoustic	Morphological	Molecular	Haplotypes	
***B*. *japonica***	Amami	Amami	NAZ	15	21	15	8	MF536314–MF536321
	Okinawa	Okinawa	OKI	14	5	15	2	MF536322, MF536323
	Yaeyama	Ishigaki	ISH	5	9	12	6	MF536324–MF536329
		Iriomote	IRI	5	4	12	8	MF536324, MF536325, MF536330–MF536335
	Northwestern TW	XinDian Stream	XD	15	9	12	6	MF536336–MF536341
		ZhongGang Stream	ZG	23	8	12	10	MF536338, MF536342–MF536350
		Wu Stream	WU	19		12	7	MF536338, MF536350–MF536355
		ZhouShui Stream	ZS	15	8	12	7	MF536350, MF536352, MF536355–MF536359
***B*. *otai* sp. nov.**	Southern TW	BaZhang Stream	BZ	18		10	5	MF536360–MF536364
		GaoPing Stream	GP	18		10	4	MF536360, MF536363, MF536365, MF536366
		DongGang Stream	DG	19	11	10	9	MF536360, MF536367–MF536374
		FongGang Stream	FG		10	10	3	MF536360, MF536374, MF536375
	Eastern TW	BeiNan Stream	BN	15		10	7	MF536376–MF536382
		Baliwan stream	BL		11			
		XiuGuLuan Stream	XG	19	11	10	8	MF536376, MF536383–MF536389
		MuGua Stream	MG	17		10	6	MF536376, MF536385, MF536390–MF536393
		MeiLun Stream	ML		8	10	4	MF536390, MF536392, MF536394, MF536395
		HePing Stream	HP	24		10	5	MF536396–MF536400
		NanAo Stream	NAO	21	8	10	6	MF536398–MF536403
		LanYang Stream	LY	19	10	10	9	MF536398, MF536404–MF536411
Total				281	133	212	98	

Based on previous literature, mitochondrial cytochrome *b* was suitable to study genetic differentiation at intraspecific level of *Buergeria* frogs [14, 19, 22]. Therefore, we amplified the complete cytochrome *b* fragment by using a pair of primers designed for *Buergeria* [14]: BueCBF1: 5’-TTTCTGCCAGGRTTYTAACCTAGACC-3’, and BueCBR7: 5’-AGTAGATTGSK GAAGCTAGTTGACC-3’. Polymerase chain reactions (PCRs) were conducted in a 20-μl reaction volume containing 1x PCR buffer (10 mM Tris-HCl, pH 9.0; 50 mM KCl; 0.01% (w/v) gelatin; and 0.1% Triton X-100), 0.8 U of Taq DNA polymerase (Amersham Bioscience, New Jersey, USA), 0.5 mM dNTP, 0.2 μM each primer, and 50 ng of template DNA. The PCR conditions consisted of denaturation at 94°C for 3 min, followed by 35 cycles of 94°C for 30 sec, 52°C for 40 sec, and 72°C for 90 sec, with a final extension at 72°C for 10 min using an iCycler Thermal Cycler (Bio-Rad). PCR products were purified with the PCR Product Pre-Sequencing Kit (USB Corp., Cleveland, OH, USA) and subsequently used as the template for direct DNA sequencing reactions with the DYEnamic ETDye Terminator Cycle Sequencing Kit (Amersham Pharmacia Biotech). Sequencing products were run on a MegaBACE 1000 automated DNA sequencer (Amersham Bioscience, New Jersey, USA). The sequences were determined in both directions, and the original signals were proofread by using SEQUENCHER 4.7 software (Gene Codes Corp., MI, USA). All the haplotypes were submitted to GenBank (MF536314 –MF536411).

### Phylogenetic analyses

Cytochrome *b* sequences from three congeners, *B*. *buergeri* (AB127977), *B*. *oxycephala* (KX021967), and *B*. *robusta* (JF802785 and JF802938), were included in phylogenetic analyses; while sequences of two rhacophorid outgroups, *Rhacophorus dennysi* (KM035412) and *R*. *schlegelii* (AB202078), were used to root the tree. The best-fit model of this dataset, comprising 104 haplotypes and 1016 bp in length, was assessed by using the Bayesian information criterion (BIC) as implemented in jModelTest 3.6 [23]. Results of this analysis suggested HKY+I+G as the best-fit substitution model, with unequal base composition, 4.4903 transition/transversion ratio, a proportion of invariable sites (I) = 0.4390, and a gamma value (G) = 0.8690. Maximum likelihood analysis was performed by using PhyML 3.0 [24], with starting trees obtained by neighbor joining (BIONJ), and NNI tree improvement. Two different approaches were used to evaluate the reliability of the inferred phylogenetic tree, including 1,000 ML bootstrap replicates implemented by PhyML 3.0, and Bayesian posterior probability conducted by using MRBAYES 3.2.2 [25]. In the latter analysis, two independent runs of 5 x 10^7^ generations with eight MCMC chains each were conducted simultaneously, starting from random trees and resampling each tree every 1000 generations. The standard deviation of split frequencies between runs (<0.01) and the effective sample size (ESS) as measured by TRACER 1.5 [26] were monitored to ensure stationarity, convergence and correct mixing of the chains and to determine the correct number of generations to discard as a burn-in for the analyses (first 20%). Converged MRBAYES runs were combined after the exclusion of burn-in, and a majority rule consensus tree was created with nodal confidence assessed by posterior probabilities. Finally, the values of statistical supports from ML bootstraps and BPPs were labelled on corresponding nodes. Alignments, tree topologies, and all further information about phylogenetic analyses in this study are available on TreeBASE at http://purl.org/phylo/treebase/phylows/study/TB2:S21450.

### Acoustic data collection

A successful record was defined as at least one minute or 10 calls obtained from each male. A total of 281 individuals were successfully recorded, from 17 drainages across 7 geographic regions (Fig 1; Table 1) during the breeding season of 2015. Advertisement calls were recorded by using a Sony ECM-CG50 directional microphone attached to a digital recorder (Sony PCM-M10) at a 44.1 kHz, 16 bit resolution. The callers were then temporarily captured to prevent from repeated sampling, and measured for snout-vent lengths (SVLs) to the nearest 0.1 mm by using a digital caliper (Mitutoyo, Kanagawa, Japan), and weighed their body masses to the nearest 0.01 g by using an electronic scale (Dr. AV Technology Co., Ltd.). Except for the type series, the frogs were released back to the original localities on the same night after all recordings were accomplished. Since ambient temperature and moisture may influence call characteristics [27], we also measured air temperature and humidity using a thermos-hygrometer (Lutron, Taipei, Taiwan). Recordings were digitized and characterized as acoustic traits by Raven Pro v1.4 (Cornell Lab of Ornithology, Ithaca, NY, USA).

### Call analyses

The acoustic signals of *Buergeria* frogs have never been scientifically quantified. In this study, *B*. *japonica sensu lato* was observed to deliver two types of signals: long calls and short calls. Each individual is able to express both types of calls, which might switch between each other pending on the density of the chorus (Y.-H. Wang and Y.-W. Hsiao, personal observation). These two types of calls could be defined by the number of pulses: calls with more than three pulses after the first rising peak were defined as long calls; while those with less than three pulses were characterized as short calls. These two types can be easily recognized by human ear, as well as from the waveforms.

We chose three temporal and two spectral parameters to analyze the short calls, which have been proposed to be associated with female choice in other Anuran [9, 28, 29]: (1) call duration (DT), the time from the beginning to the end of a call; (2) call rise time (RT), the time from the beginning to the highest peak on the waveform of a call; (3) call fall time (FT), the time from the highest peak on the waveform to the end of call; (4) dominant frequency (DF, in Hz), the highest peak reached by a call on the power spectrum (FFT = 1024 points, Hann window); and (5) interquartile range (IQR), calculated by subtracting the 3^rd^ quartile value from the 1^st^ quartile value of frequency bandwidth of a call. Call parameters of each individual were obtained by averaging the values of 10 calls from the same caller. In order to standardize the impact of external environments, we summarized air temperature and humidity by principal component analysis (PCA), then chose PC1 as the “ambient factor”. To test differences by call properties among geographical regions, we used general linear mixed model (GLMM) in which regions were treated as a fixed factor and sample localities were nested within the regions as a random factor. We included “ambient factor” as a covariate. The GLMM was performed using SAS 9.4 statistic software (SAS Institute Inc., Cary, NC).

On the other hand, long calls are constituted by a series of complicated components, which makes them difficult to be quantified. First we categorized the long calls into three different types (Type 1A from Japanese clade, Type 1B and Type 2 from Taiwanese clade; see “Results” for details). We identified the type(s) produced by each frog, and conclude the ratio of frogs which could deliver either type(s) from each sampling locality. To access the association between long call types and regions, we used chi-square test and computed adjusted standardized residuals for each cell of contingency table.

### Morphological data collection

A total of 133 adult male frogs were collected from 14 populations (Table 1). Frogs were euthanized by absorbing lethal dose of benzocaine through their skin, following the standard protocol approved by IACUC, National Taiwan Normal University (license No.104033 and No.106024). We took 19 morphometric measurements followed previous studies [30, 31, 32, 33, 34], including: (1) snout-vent length (SVL), the length between the tip of the snout to the cloaca; (2) head length (HL), the length between tip of snout to the posterior edge of jaw angle; (3) head width (HW), the maximum width of the head on the level of mouth angles in ventral view; (4) eye length (EL), the length between the anterior and posterior corners of the eye; (5) interorbital distance (IO), the shortest length between the medial edges of eyeballs in dorsal view; (6) intercanthal distance (IC), the length between the front edge of the eyes; (7) eye-nostril length (END), the length between the anterior corner of eye and the nostril center; (8) width of upper eyelid (UEW), the widest length from the medial edge of eyeball to the lateral edge of the upper eyelid; (9) forelimb length (FLL), the length from the tip of the third finger to the end of the forelimb armpit; (10) forelimb arm length (FAL), the length from the forelimb armpit to the elbow; (11) hand length (HAL), the length from the proximal end of outer metacarpal tubercle to the tip of the third finger; (12) first finger length (1FL), the length from the first fingertip to the base of the finger; (13) third finger length (3FL), the length from the third fingertip to the base of the finger; (14) hindlimb length (HLL), the length of straightened hind limb from groin to tip of the fourth toe; (15) tibia length (TL), the length between the knee and tibiotarsal articulation; (16) tarsus length (TTL), the length between tibiotarsal articulation and the fourth toe; (17) inner metatarsal tubercle length (iMTL), the maximal diameter of inner metatarsal tubercle; (18) first toe length (1TL), the length from the end to first toe tip to the base of the toe; and (19) forth toe length (4TL), the length from the end to fourth toe tip to the base of the toe. Webbing formula followed [35], and modified by [36]. All measurements were taken by Y.-H. Wang with a digital caliper and recorded to the nearest 0.1 mm (Mitutoyo, Kanagawa, Japan). Type specimens for morphological measurements were deposited in National Museum of Natural Science (NMNS), Taichung, Taiwan.

### Morphology analyses

First, we examined the association between SVL and regions in order to evaluate the possible size differences across different island groups. The remaining 18 traits (from 2^nd^ to the 19^th^) were divided by SVL in order to standardize these measurements and were used in statistical analyses. To reduce the number of variables, we input all parameters into principle component analysis (PCA), and used the first six principal components with eigenvalues greater than 1 as new variables in the subsequent discriminant analysis (DA) and general linear mixed model (GLMM). We used phylogenetic analyses to establish groups, and evaluate the congruency of individual assignments by using discriminant analysis. We chose the grouping criteria from two to six based on genetic lineages, and used GLMM to test among-group differences of these principal components summarized from morphological characters.

### Behavioral response to conspecific and heterospecific calls

In order to test the behavioral response of a frog toward conspecific or heterospecific males, we conducted playback experiments in a contact zone from southwestern Taiwan, where the two clades are parapatrically distributed with an extremely narrow transition border. Genetic analysis using mitochondrial sequences indicated that the northern population (Beigang Stream, Gukeng Township) belongs to Japanese clade, while the southern population (Niuchou Stream, Zhuqi Township) belongs to Taiwanese clade. The two localities were spaced with only 8.9 km by low-elevation hardwood forests.

When a male frog was targeted in the chorus, its spontaneous call was recorded for one minute and was regarded as pretreatment. Three types of long calls were then provided to the target male: (1) Type 1A from Japanese clade; (2) Type 1B from Taiwanese clade; and (3) Type 2 from Taiwanese clade. A speaker was placed 0.5 m from the target male, which simulated the situation when a competitor was provoking the target male. The target male would express territorial calls to response the intruder, which were simultaneously recorded by a directional microphone placed 0.5 m from the frog (with a 90° angle from the speaker). The three types of calls were played in a random order, lasted for one minute for each type, and spaced for one minute between each other. A trial was defined to be successful when the target male response to anyone of the three types for more than 20 territorial calls within any of the 1-min interval.

We used three calculations to evaluate the diagnostic ability of the target male. First, we calculate the net number of territorial calls when exposed to each treatment, which was defined as “Response call numbers”. However, owing to the variation of defensiveness among males (some individuals showed stronger/weaker responses toward the intruder than the others), we further standardize the response of each target male by calculating the percentage of calls allocated to response each kind of intruders. This was defined as “Response call ratio”, representing the energy of allocation toward each type of intruder. From these two evaluations, we were able to figure out a call type which could most effectively provoke the target male. This was defined as the “Highest defense opponent” of each individual. These results were compared to the null hypothesis (no preference among the three types) by using chi-square tests.

## Results

### Deep genetic divergence between Japanese and Taiwanese clades

**Fig 2:**
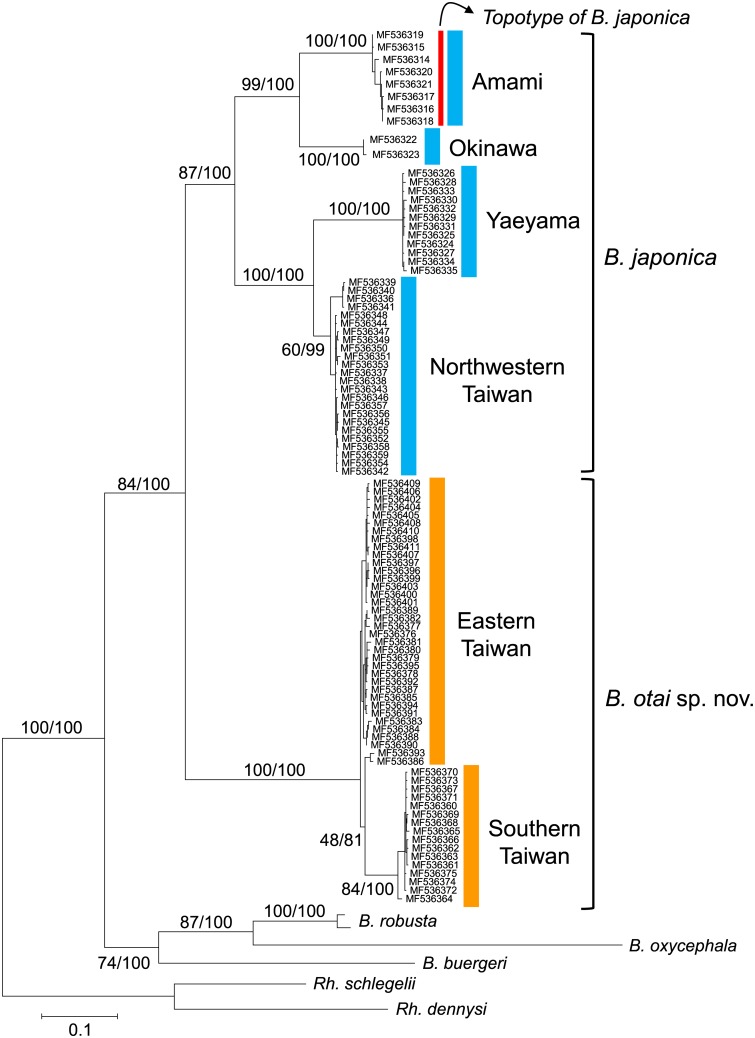
Maximum likelihood phylogeny of *Buergeria japonica sensu lato*. Phylogenetic tree was constructed by using PhyML 3.0 based on complete cytochrome *b* sequences, with 1000 bootstrap replicates and Bayesian posterior probability as statistic supports. Japanese clade (blue) and Taiwanese clade (orange) represent 16.19% between-group sequence divergence (*p*-distance).

Maximum likelihood and Bayesian trees represented precisely identical topology for the phylogenetic relationship, as represented in Fig 2. *Buergeria japonica sensu lato* comprises two distinctly divergent clades (Fig 2): one comprises four allopatric lineages from Amami (the type locality of *B*. *japonica*), Okinwan, Yaeyama, (Ishigaki and Iriomote), and northwestern Taiwan. The other comprises two lineages distributed in eastern and southern Taiwan, respectively. Hereafter we define the former as Japanese clade, and the latter as Taiwanese clade. The mean divergence between the two major clades reached 0.1619, with a maximum of 0.1786 (Amami vs. southern Taiwan). Divergences among the four lineages within Japanese clade are still large, ranging between 0.0890 and 0.1456; and the divergence between the two lineages with Taiwanese clade is 0.0358. Variation within each lineages does not exceed 0.0066 (Table 2).

**Table 2:** Within-clade (diagonal) and between-clade (upper-right) divergences among the four major lineages in the Japanese clade (*Buergeria japonica*) and two major lineages in the Taiwanese clade (*Buergeria otai* sp. nov.).

Species	Clade	*Buergeria japonica*				*Buergeria otai* sp. nov.	
		Amami	Okinawa	Yaeyama	NW Taiwan	S Taiwan	E Taiwan
*B*. *japonica*	Amami	0.0055	0.0890	0.1456	0.1333	0.1768	0.1716
	Okinawa		0.0005	0.1420	0.1253	0.1612	0.1605
	Yaeyama			0.0019	0.0765	0.1687	0.1642
	NW Taiwan				0.0059	0.1552	0.1561
*B*. *otai* sp. nov.	S Taiwan					0.0020	0.0358
	E Taiwan						0.0066

### Rapid short calls and a novel type of long call in Taiwanese clade

**Fig 3:**
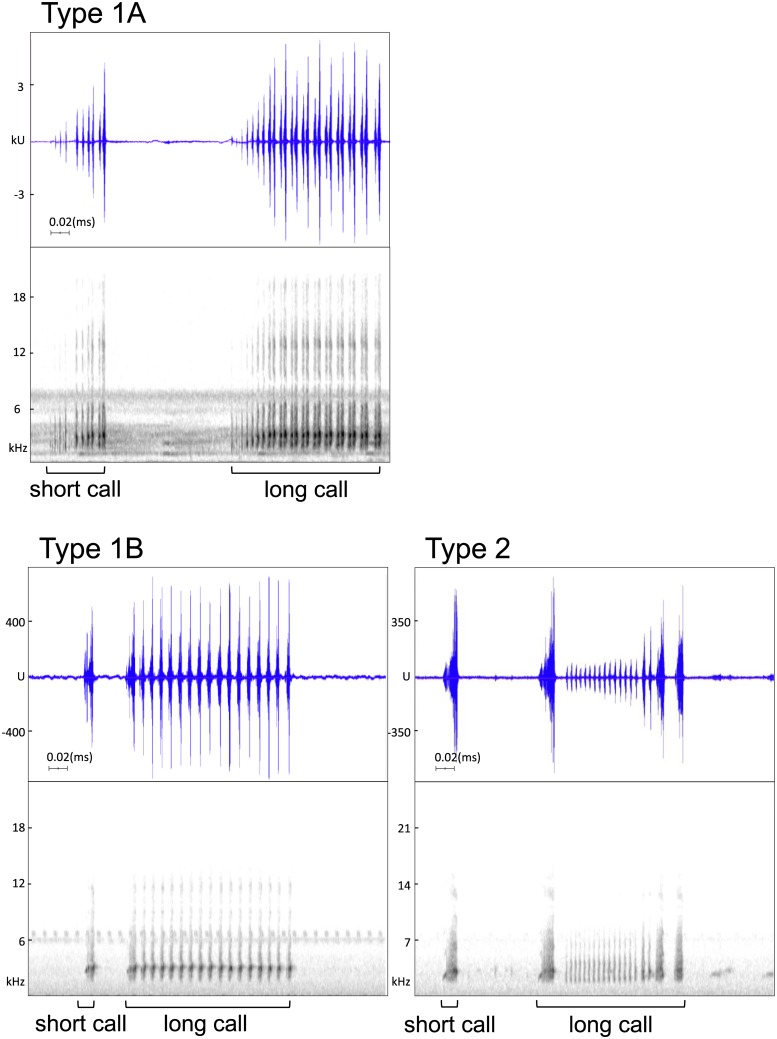
Comparison of long call sonograms between the Japanese and Taiwanese clades. The Japanese clade (*Buergeria japonica*) could express only one type of long call (Type 1A), yet the Taiwanese clade (*Buergeria otai* sp. nov.) is capable to express an additional type (Type 1B and Type 2). The units (u or ku) displayed on the vertical axis of a waveform view are the actual sample values in the signal, which are proportional to the sound pressure at the microphone when the sound was recorded (Raven Pro v1.4, Cornell Lab of Ornithology, Ithaca, NY, USA). Sonograms in this figure: Type 1A: No. 150518_03#2 from Wu Stream, northwestern Taiwan; Type 1B: NMNS 19824 (paratype of *B*. *otai* sp. nov.) from Donggang Stream, southern Taiwan; Type 2: NMNS 19816 (paratype of *B*. *otai* sp. nov.) from Donggang Stream, southern Taiwan.

Diagnosable long calls were recognized from 236 individuals (84% among the totally 281 sound records). In the northwestern Taiwan and Ryukyus (Japanese clade), frogs could deliver only one type of long call (Type 1); whereas frogs from eastern and southern Taiwan (Taiwanese clade) are able to express two different types (Type 1 and Type 2; sonograms see Fig 3 and S1 Fig). Type 1 was characterized by a serial of regular, long and lasting consecutive pulses with similar amplitude, with a most common pulse number on 16 or 17. Although with very similar sonograms, Type 1 from the two clades represent slight temporal and structural differences and was further named as Type 1A for Japanese clade (S1 and S2 Audios) and 1B for Taiwanese clade (S3–S6 Audios). In contrast, Type 2 long call (unique for eastern and southern Taiwan) starts with a major peak at the beginning, followed by a serial of vary rapid pulses from weak to strong, and usually ends with another short peak (S7–S10 Audios).

Call type usage was strongly associated to the geographic distribution (χ^2^ = 185.5, d.f. = 6; *p*<0.0001). All frogs from Amami, Okinawa, Yaeyama, and northwestern Taiwan produced exclusively Type 1A long calls (Fig 1B). In contrast, many frogs from eastern and southern Taiwan were heard to express the two types of long calls simultaneously in their sound records (therefore, the sum of usage percentage exceed 100% in Fig 1B and 1C). Type 2 was obviously the dominant call which was more frequently emitted by most populations (only one exception) in this region (Fig 1C).

**Fig 4:**
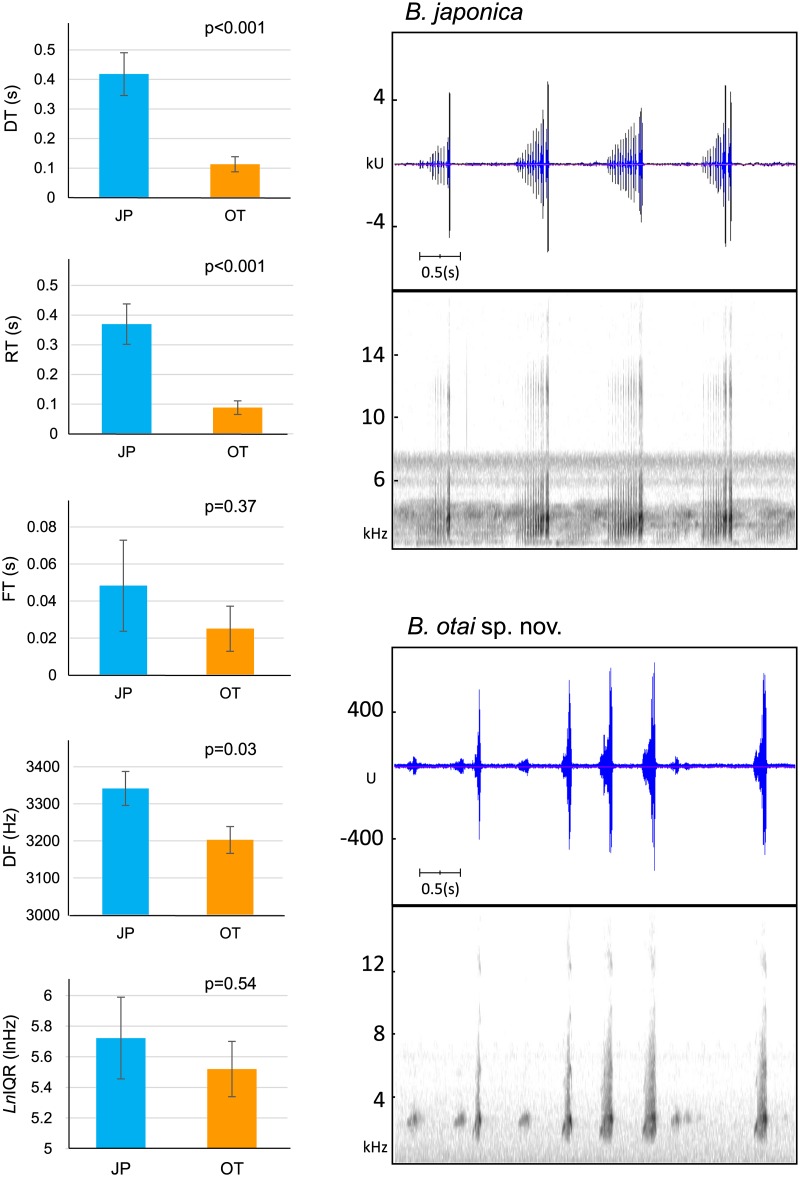
Comparison of short call sonograms between the Japanese and Taiwanese clades. The Japanese clade (*Buergeria japonica*, abbreviated as JP) expresses calls with a comparatively slower tempo, which was represented by their significantly higher call duration (DT, in sec; F_1,13_ = 20.75, *p*<0.001) and call rise time (RT, in sec; F_1,13_ = 20.26, *p*<0.001). On the other hand, the Taiwanese clade (*B*. *otai* sp. nov., abbreviated as OT) expresses a lower dominant frequency (DF, in kHz; F_1,13_ = 5.59, *p* = 0.03). Call fall time (FT) and interquartile range (IQR) were not significantly different the two clades. The units (u or ku) displayed on the vertical axis of a waveform view are the actual sample values in the signal, which are proportional to the sound pressure at the microphone when the sound was recorded (Raven Pro v1.4, Cornell Lab of Ornithology, Ithaca, NY, USA). Sonograms in this figure: *B*. *japonica*: No. 150518_03#2 from Wu Stream, northwestern Taiwan; *B*. *otai* sp. nov.: NMNS 19816 (paratype) from Donggang Stream, southern Taiwan.

Analyzable short calls (more than 10 continuous repeats) were obtained from 251 individuals (89% among the totally 281 sound records). Owing to the small valid sample size, the Okinawa population (n = 2) was excluded. Among the five characters being evaluated from short calls, call duration (DT; GLMM, F_1,13_ = 20.75, *p*<0.001), call rise time (RT; F_1,13_ = 20.26, *p*<0.001), and dominant frequency (DF; F_1,13_ = 5.59, *p* = 0.03) of short calls were significantly different (Fig 4). Call duration (DT) of Taiwanese clade (mean ± SD = 0.1127 ± 0.0258 sec) was significantly shorter than Japanese clade (0.4180 ± 0.0725 sec). Call rise time (RT) represent a similar pattern, where the Taiwanese clade (0.0882 ± 0.0228 sec) was significantly shorter than their Japanese sibling (0.3697 ± 0.0682 sec). On the other hand, the two clades are also distinguishable by a significantly different dominant frequency (DF): 3202.7 ± 36.2 Hz for Taiwanese clade, and 3341.5 ± 46.1 Hz for Japanese clade. This means that Taiwanese clade expresses a rapid, condense, and depressing tempo; but with a sound of lower tone. Call fall time (FT) and interquartile range (IQR) were not significantly different the two clades. Ambient factors, SVL, and body mass had no effect on any short call properties (p>0.05 in all characters).

### Larger head dimension and small regular spots on thighs in Taiwanese clade

**Fig 5:**
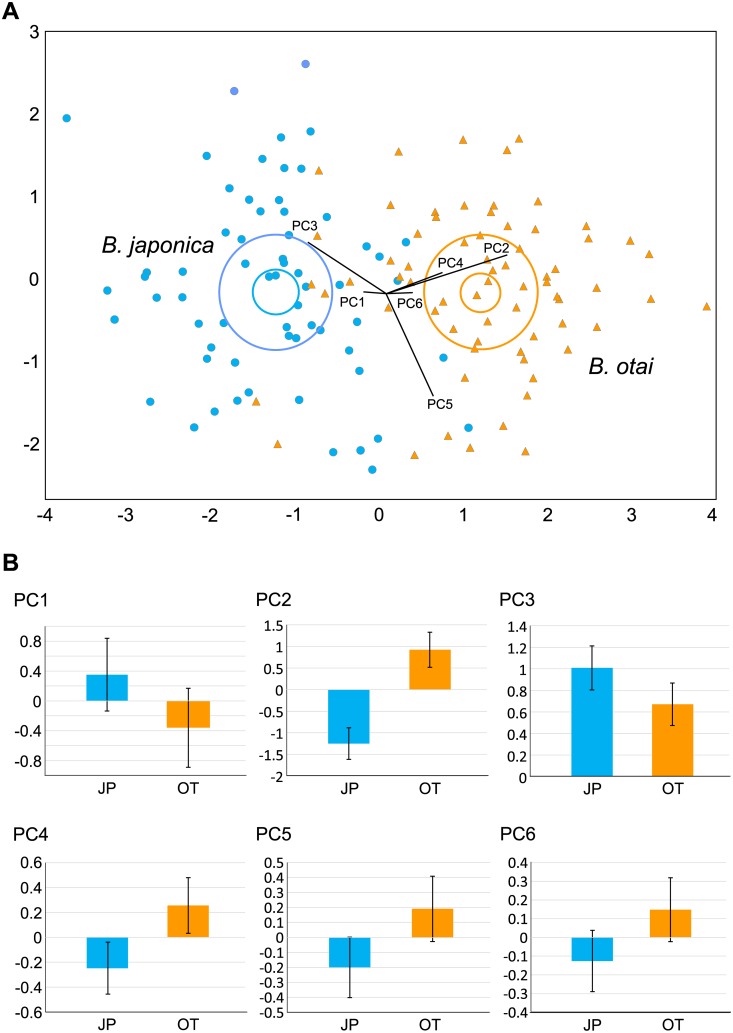
Principal component analysis and morphological differentiation between the Japanese and the Taiwanese clades. The Japanese clade (*Buergeria japonica*) is abbreviated as JP and represented in blue; and the Taiwanese clade (*Buergeria otai* sp. nov.) is abbreviated as OT and represented in orange. These two clades differed significantly in PC2, which was consisted majorly by the size and shape of the head (F1,14 = 15.85, p = 0.001).

Based on the results of PCA (Fig 5A), we included six principal components (from PC1 to PC6) as new variables and used them in discriminant analysis. According to the phylogeny, we tried to discriminate the regions by 2, 3, 5, or 6 clusters. The 2-cluster approach yielded to the best discrimination results, with concordant to their genetic and acoustic differentiation. Such a cluster division correctly assigned 89.8% of individual to a cluster, higher than the 3-cluster approach (86.7%), 5-cluster approach (81.3%), and 6-cluster approach (70.7%). The individuals in the two clusters differed significantly in PC2 (Fig 5B), which was consisted majorly by the size and shape of the head (F_1,14_ = 15.85, *p* = 0.001). In this analysis, Taiwanese clade has a significantly larger head dimension compared to Japanese clade.

**Fig 6:**
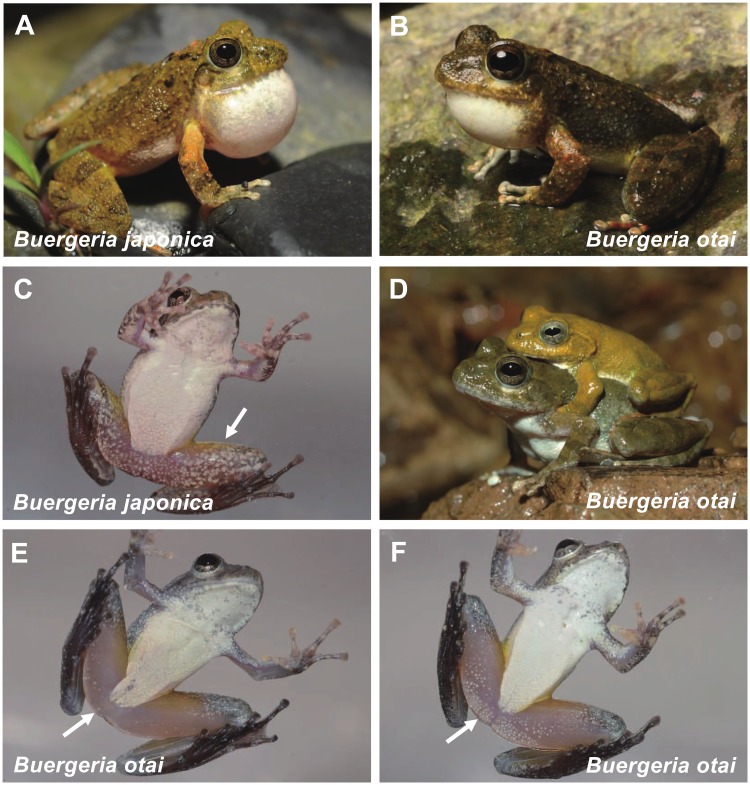
*Buergeria japonica* (A, C) and *Buergeria otai* sp. nov. (B, D, E, F) in live. The irregular patches of *B*. *japonica* on the thighs could be compared to the regular tiny spots of *Buergeria otai* sp. nov., regardless the intraspecific variation from very few (E) to many (F). Photographed by YJ Liang (A, C), CM Tsao (B), and HN Nguyen (D, E, F).

On the other hand, we also discovered a diagnostic character which helps to distinguish between the representatives of the two clades in life. The ventral view of thighs in Japanese clade is covered by irregular white patches with some variation in coverage ratio (Fig 6C). In contrast, Taiwanese clade shows condense, tiny, and white spots in a regular size and shape. Although the number and density of these spots represent high variation from none (Fig 6E) to many (Fig 6F), most of the spots concentrate on the base of the thighs.

### Strongest response to conspecific long calls

**Fig 7:**
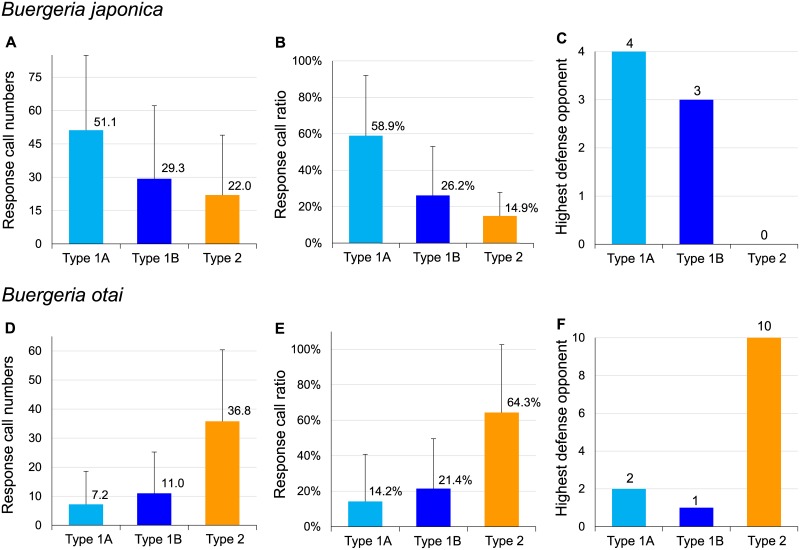
Behavioral response of male frogs toward conspecific and heterospecific calls. Both species expressed higher defensiveness against conspecific rather than heterospecific calls. *Buergeria japonica* expresses highest “Response call numbers” (A) and “Response call ratio” (B) toward Type 1A long call; while *Buergeria otai* sp. nov. expresses highest response to Type 2 (D and E). Conclusively, both species choose the conspecific call as their “Highest defense opponent” (C and F). It is noteworthy that *B*. *japonica* is not able to distinguish between Type 1A (conspecific) and Type 1B (heterospecific) owing to the similarity.

Males from both clades showed stronger defensiveness against conspecific than heterospecific calls. The northern population (belonging to Japanese clade) showed strongest response to Type 1A calls (mean = 51.1 calls in the 1-min interval, Fig 7A), which represented 58.9% energy allocation (Fig 7B). The southern population (Taiwanese clade) showed strongest response to Type 2 calls (mean = 36.8 calls, Fig 7D), which represented 64.3% energy allocation (Fig 7E). Ten among the thirteen southern males chose conspecific call (Type 2) as the highest defense signal (Fig 7F), indicating that this acoustic signal was used by the males to distinguish their opponents. On the other hand, although Type 1A call received the highest response call numbers and response call ratio, and was chosen as the highest defensive opponent by the northern males, three among the seven individuals made an incorrect decision to defense against Type 1B which was delivered by the heterospeific males (Fig 7C). These results suggested that some of the males from Japanese clade were not able to distinguish between Type 1A and Type 1B calls, possibly due to their similarity in temporal and energy traits. Conclusively, males from both clades use long calls as diagnostic signal, and showed a strong tendency to response against conspecific calls.

## Discussion

The taxonomic status of *Buergeria japonica* has been proposed to be questionable by two recent studies based on molecular evidence [19, 22]. In this study, we further used three additional criteria: acoustic signal analysis, morphological characters, and behavioral experiments, to provide further evidences on the species status of the two clades revealed within *B*. *japonica sensu lato*. Results from all these criteria are consistent with molecular evidence, supporting the splitting of *B*. *japonica* into two species: one comprising populations in the Ryukyu Archipelago and northwestern Taiwan (Japanese clade), and the other including the remaining populations from eastern and southern Taiwan (Taiwanese clade). Both short and long calls are significantly different between the two species; Taiwanese clade delivers their short calls in a faster tempo, and they also express a comparatively lower frequency. Most importantly, this species is able to express a second type of long call (Type 2), which was never recorded from their sister clade.

Although evolved to represent prominent molecular and acoustic divergences, morphology remains similar between the two species. The Taiwanese clade can be distinguished by a larger PC2, which was contributed mainly by the size of the head (Fig 5). Furthermore, markings on the ventral side of the thighs, regular tiny spots in Taiwanese clade versus irregular light patches observed in Japanese clade, also helps to delimitate the two species (Fig 6). Nevertheless, these morphological differences are obscure compared to their diagnostic acoustic differentiation. Similar situation has been well addressed in the greenish warbler *Phylloscopus* complex, which are almost indiscernible through morphology but display distinct acoustic signals [37, 38]. Two recent cases in Southern Asia came from the morphologically similar *Mircohyla ornata* species complex (Microhylidae) and several *Theloderma* species (Rhacophoridae), where cryptic species were described by using molecular and acoustic characteristics [39, 40, 41].

Acoustic signal plays a key role in pre-mating isolation. Referring to Paterson’s Recognition Species Concept (RSC) [42, 43], the differentiation of advertisement calls gave the primary line of evidence to suggest the biological identity in this candidate species. The last piece of puzzle came from the play-back experiments, which demonstrated that these frogs are capable of using long calls as species recognition signals. Japanese clade responses most intensively against Type 1, while Taiwanese clade responses against Type 2. However, some of the individuals in the Japanese clade were not able to distinguish between Type 1A (conspecific) and Type 1B (heterospecific) owing to their acoustic similarity. This phenomenon partially explained the preliminary observation that frogs near the contact zone tend to use the type which represent their own characteristics. A recent study in the southwestern contact zone (Hsiao *et al*., unpublished data) substantiated the probable phenomenon of “reproductive character displacement” and “reinforcement”, which were proposed as the central hypotheses to explain how sister species evolve to maintain their genetic integrity [44, 45, 46]. This inference requires intense tests throughout the island, especially across the contact zones.

Based on the four lines of evidences, Japanese and Taiwanese clades are distinctly differentiated by their genetic integrity, acoustic differentiation, stable diagnostic morphological traits, and behavioral discernment. Referring to the type locality in Amami Island, Ryukyus [47], *B*. *japonica* should be restricted to populations distributed from Ryukyu Archipelago to the northwestern region of Taiwan, whereas populations on the eastern and southwestern regions of Taiwan should be recognized as a yet undescribed species. Herein we describe the latter as a new species as below.

## Species description

**Family Rhacophoridae Günther**, **1859**

**Genus *Buergeria* Tschudi**, **1838**

### *Buergeria otai* sp. nov.

#### Synonymy

*Ixalus japonicus*—Hallowell, 1861 "1860", Proc. Acad. Nat. Sci. Philadelphia, 12: 501. *Polypedates japonicus*—Stejneger, 1907, Bull. U.S. Natl. Mus., 58: 155. *Rhacophorus (Rhacophorus) japonicus*—Ahl, 1931 in Das Tierreich, 55: 111. *Rhacophorus (Rhacophorus) buergeri japonicus*—Wolf, 1936, Bull. Raffles Mus., 12: 166. *Buergeria japonica*—Liem, 1970, Fieldiana, Zool., 57: 90.

#### Holotype

**Fig 8:**
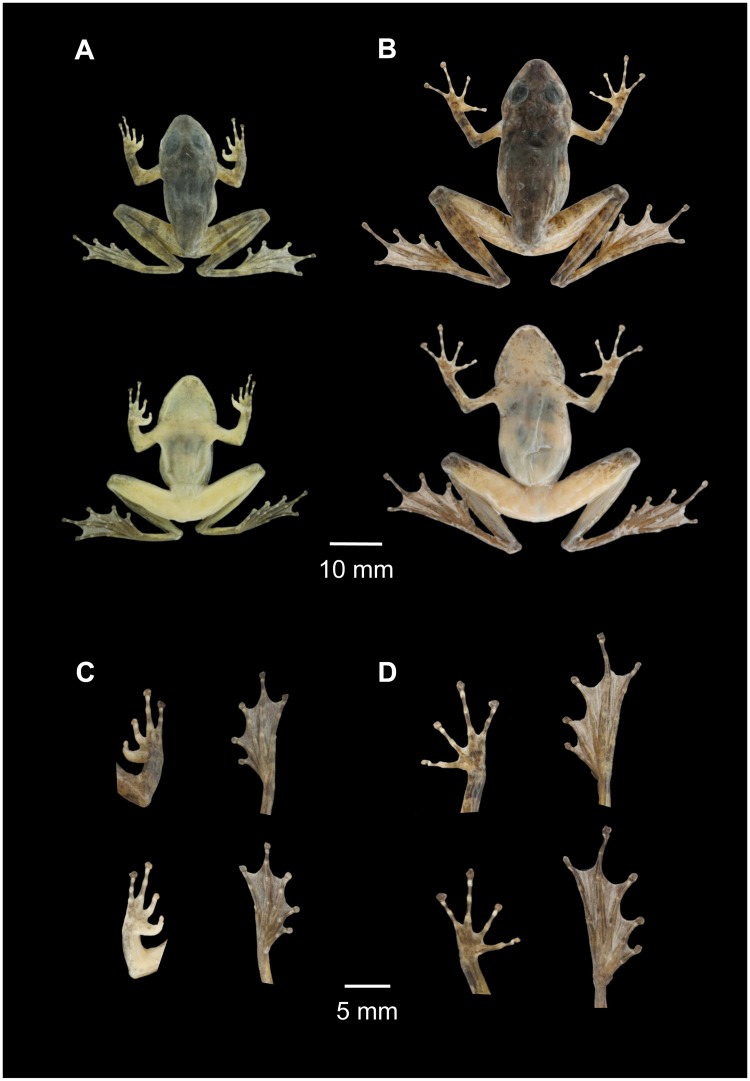
Holotype (A and C, male, NMNS 19819) and one of the paratypes (B and D, female, NMNS 19871) of *Buergeria otai* sp. nov.

NMNS 19819 (Fig 8; S1 Fig; S3 Audio), adult male collected by Ying-Han Wang and Chia-Wei Lu on July 14th, 2015 from Taiwan, Pingdong County, Donggang Stream (22.626340 N,120.643342 E) with elevation ca. 90 m. The frog was found forming a chorus with approximately 50 other males on a temporary shallow shoal beside a mountain stream with 5 meters in width. The environment of type locality is flow land and ditches surrounded by hardwood forests. Audio file of the acoustic signal produced by the holotype is available online (S1 Fig; S3 Audio).

#### Paratypes

Twenty-two males and five females collected from southern and eastern Taiwan. Males: NMNS 19815, 19817, 19822, 19824 collected from Donggang Stream, Pingdong County; NMNS 19825, 19831, 19833 from Fonggang Stream, Pingdong County; NMNS 19856, 19859, 19865 from Reisui, Hualien County; NMNS 19846, 19850, 19853 from Baliwan Stream, Hualien County; NMNS 19788, 19799, 19803 from Meilun Stream, Hualien County; NMNS 19806, 19808, 19812 from Nanao Stream, Yilan County; and NMNS 19838, 19840, 19844 from Lanyang Stream, Yilan County. Five females: NMNS 19867, 19868, 19869 from Meilun Stream, Hualien County; and NMNS 19870, 19871 (Fig 8) from Nanao Stream, Yilan County. All the above specimens were collected in 2015 by Ying-Han Wang, Ko-Huan Lee, Jhan-Wei Lin, Jia-Wei Lu, Wen-Hsuan Tseng, Yu-Wei Hsiao, Wei-Yi Tsai, and Yi-Long Li and are now preserved in National Museum of Natural Science (NMNS), Taichung, Taiwan. Audio files of some among these individuals are available online (S1 Fig; S4–S10 Audios).

#### Etymology

The specific epithet of the new species “*otai*” is a latinized patronymic noun in genitive case, dedicated to Prof. Hidetoshi Ota for his great contribution to herpetology and biogeography in East Asia, including Taiwan and adjacent regions. During 1980s to 1990s, Ota published four reptile species in Taiwan, carefully reviewed the herptile fauna across the East Asian Arc, and provided great assistance and encouragement to new-generation herpetologists in this region. We suggest the following common name “Ota’s stream tree frog” in English.

#### Diagnosis

*Buergeria otai* sp. nov. is characterized by a combination of the following characters: (1) a small-sized rhacophorid, body moderately slender; (2) SVL in adult males 23.1–29.3 mm (N = 133; mean ± SD = 26.57 ± 1.21 mm); females 29.7–37.5 mm (N = 3; 32.44 ± 4.42 mm); (3) dorsum slightly tubercular, with a pair of parallel tubercles on scapula; (4) head triangular, snout rounded and somewhat acute; (5) tips of fingers and toes dilated, forming expanded disks (over twice the width of phalanges); (6) tibiotarsal articulation on adpressed limb reaching beyond snout tip; (7) forelimb webbing absent; (8) hindlimb webbing partial, webbing formula (the number of phalanges free of web): I 1–1$\frac{1}{3}$ II 1–2 III 1–1$\frac{1}{2}$ IV 2–1 V; (9) vertebral stripe absent; (10) dark dorsal marking in the shape of inverted triangle between the eyes; (11) dark dorsal marking in a form of letter X or H extending from scapula to the middle of the back; (12) chin gray-white, with small irregular mottling; belly gray-white; (13) arms and thighs with sparse brownish bands; (14) regular tiny white spots on the ventral side of the thighs; usually concentrated at the base of the thighs.

#### Measurements of holotype (in mm)

SVL 26.7; HL 10.4; HW 10.1; EL 4.1; IO 2.5; IC 4.4; END 2.6; UEW 2.6; FLL 19.9; FAL 6.9; HAL 7.9; 1FL 2.1; 3FL 4.6; HLL 52.2; TL 16.6; TTL 21.6; iMTL 1.3; 1TL 3.0; 4TL 10.4.

#### Description of holotype

Mature adult male, SVL 26.7 (all measurements in mm); moderately slender, body elongated. Head triangular, as wide as body; slightly longer (HL/HW = 103.0%). Snout comparatively long (22.8% HL), triangular in dorsal view with a slightly obtuse snout, moderately pointed in profile. Canthus rostralis distinct; loreal region weakly concave; nostril round, lateral, below canthus rostralis, closer to tip of snout (10.9% HL) than to eye (25.7% HL). Eye moderate sized, EL 39.4% of HL, larger than eye—nostril distance (25.7% HL); interorbital distance 24.0% of HL, 24.8% of HW. Interorbital space flat. Tympanum barely visible, rounded; diameter 46.3% of eye length; tympanic annulus inconspicuous. Supratympanic fold prominent, extending from posterior to eye to posterior margin of arm insertion. External vocal sac subgular, single, comparatively large.

Forelimbs moderately long; FAL 25.8% SVL; HAL 29.6% SVL, FAL 87.3% HAL. Fingers slender, free of webbing, rounded in cross-section, no skin fringes on fingers present. First finger well-developed, tip slightly expended. Tips of fingers II, III, IV flattened, bearing medium-sized discs; width of discs on Finger II, III, and IV represent 135%, 205%, and 243% of the thinnest diameter of the middle phalanx. Relative finger lengths: I < II < IV < III; length of finger I (2.1) 45.7% of finger III (4.6). Inner and outer metatarsal tubercles absent; subarticular tubercle prominent, rounded, single tubercle per digit.

Hind limbs relatively long, TL 62.2% of SVL, TTL 80.9% SVL. Toes long, thin, tips of toes flattened, all bearing medium-sized discs; width of discs on Toe I, II, III, IV, V represent 180%, 208%, 253%, 193%, 178% of the thinnest diameter of the middle phalanx. Relative toe lengths: I < II < III < V < IV; length of toe I 28.8% of finger IV. Webbing partial, reaching penultimate tubercles: one phalanx at toe I, one and two thirds of phalanges at preaxial side of toe II, one phalanx at postaxial side of toe II, two phalanges on preaxial side of toe III, one phalanx on postaxial side of toe III, two and a half of phalanges on preaxial side of toe IV, two phalanges on postaxial side of toe IV, one phalanx on toe V (webbing formula I 1–1$\frac{1}{3}$ II 1–2 III 1–1$\frac{1}{2}$ IV 2–1 V). Inner metatarsal tubercle ovoid-shaped, present at base of first toe; outer metatarsal tubercle absent; subarticular tubercles prominent, rounded. Dorsum slightly tubercular, with a pair of parallel tubercles on scapula.

#### Coloration in life

Dorsal coloration in life from grey to light yellow-brown. Brownish dorsal marking in the shape of inverted triangle between the eyes; in a form of letter X or H extending from scapula to the middle of the back. Sparse brownish bands on dorsal surfaces of forelimbs and hindlimbs. Reddish blotches sometimes present on dorsum or limbs. Chin gray-white, with small irregular mottling; belly gray-white. Regular tiny white spots on the ventral side of the thighs; usually concentrate at the base of the thighs.

#### Coloration in preservative

In preservative the pattern described above does not change obviously, however colors significantly faded. Dorsal background color looks dark brown; dark dorsal marking of inverted triangle between the eyes and letter X on the back faded in alcohol. Yellowish and reddish blotches fade the most; inverted triangular marking between the eyes and X-shaped dark marking on dorsum obscure; brownish cross bands on limbs retained. Ventral side looks similar, but reddish blotches totally disappeared.

#### Variation and sexual dimorphism

Individual coloration changes pending on background or light condition. Resting frogs in daytime usually have grayish color. In contrast, individuals in a chorus at night may range from gray, yellow, gold, light brown, or dark orange. In some occasions, the X- or H-shaped fades and is obscure.

Mature females (29.7–37.5 mm, N = 3) almost always larger than males (23.1–29.3 mm, N = 133). Males are characterized by the enlarged thumb at the base. During amplexus, females usually turn brownish.

#### Comparisons

*Buergeria japonica* and *Buergeria otai* sp. nov. can be easily distinguished from all the other three congeners by a much smaller body size. Males of mature *B*. *buergeria*, *B*. *robusta*, and *B*. *oxycephala* exceed 37, 42, and 34 mm, respectively; yet male *B*. *japonica* and *Buergeria otai* sp. nov. are not recorded to exceed 32 mm. Similar size difference is also prominent in females: *B*. *buergeria*, *B*. *robusta*, and *B*. *oxycephala* are not smaller than 49, 59, and 60 mm, respectively; while female *B*. *japonica* and *Buergeria otai* sp. nov. do not exceed 38 mm. *Buergeria japonica* and *Buergeria otai* sp. nov. can be further distinguished from other congeners for weak and obscure temporal folds, absence of forelimb webs, and enlarged X-shaped or H-shaped marking between forelimbs. In the wild, body shape and the dorsal marking are the easiest diagnostic features between these two species and their sympatric congener *B*. *robusta*.

Although *Buergeria otai* sp. nov. has a significantly bigger head dimension than *B*. *japonica* (Fig 5), it is more reliable to discriminate the two species by using (1) markings on the ventral side of the thighs; and (2) acoustic signals. The ventral aspect of thighs in *Buergeria otai* sp. nov. shows tiny white spots in a regular size and shape. Although the number and density of these spots represent high within-species variation from none (Fig 6E) to many (Fig 6F), most of the spots concentrate on the base of the thighs. In contrast, ventral sides of thighs in *B*. *japonica* is almost fully covered by irregular white patches (Fig 6C).

*Buergeria japonica* could express only one type of long calls (Type 1A), but *Buergeria otai* sp. nov. could express two (Type 1B and Type 2). Types 1A (S1 and S2 Audios) and 1B (S3–S6 Audios) were similar in sonograms, and were characterized by a serial of regular, long and lasting consecutive pulses with similar amplitude, with a most common pulse number on 16 or 17. In contrast, Type 2 long call starts with a major peak at the beginning, followed by a serial of rapid pulses from weak to strong, and usually ends with another short peak (S7–S10 Audios). Typical calls of *Buergeria otai* sp. nov. are usually constituted by a combination of dominant Type 2 with less dominant Type 1B calls, and are easily distinguished from *B*. *japonica* in the wild. Experienced observers even can recognize the Type 1A and 1B calls, and distinguish between the short calls of the two species based on their tempo and audio frequency. *Buergeria otai* sp. nov. has significantly shorter, faster, and more rapid short calls with a lower tone (Fig 4); but they have a slower Type 1B compared to 1A in *B*. *japonica*.

#### Distribution

*Buergeria otai* sp. nov. is distributed widely throughout all the low elevation streams of eastern and southern Taiwan with elevation below than 1500 m. The north limit of the new species in western Taiwan was recently located in Pozi Stream, while the north limit on the eastern side of the island was identified in Lanyang Stream (Y.-W. Hsiao and Y.-H. Wang, personal observation). *Buergeria otai* sp. nov. might be the most abundant species among all the 14 rhacophorids reported for Taiwan; there are huge populations especially in the eastern side of the island.

#### Natural history notes

Although belonging to the Old-world treefrog family Rhacophoridae, *Buergeria otai* sp. nov. is specialized to live in the streams like all its congeners. They prefer to gather in small ditches or shallow waters near by the streams, but seldom entering into the major river course. Breeding season usually lasts from February to October [48], with a major peak from April to July (personal observation in this study), but may appear all year round in some habitats [48]. Males gather to form chorus beside the streams after sunset, and the chorus reach its climax near midnight. Eggs 1.2–1.4 mm in diameter, attached on vegetation or spread on the substrates in shallow water, hatched after 24–36 hr. Tadpoles herbivorous or detritivorous, live benthically in shallow waters, with a larval stage period 15–30 days, depending on the water temperature.

Both *Buergeria otai* sp. nov. and *B*. *japonica* are well known for their special tolerance in geothermal hot springs, which seems to be an extraordinary adaptation from all anuran species in the world [18, 49]. The tadpoles of the frogs often show thermal affinity by approaching hot waters with temperature higher than 30°C, and their critical thermal maxima could reach more than 41°C. This adaptation was deduced to extend the breeding season, decrease the hatching rate, and increase the tadpole size [48, 50]. Furthermore, *B*. *japonica* was also well addressed for their special to salt tolerance. Although *Buergeria otai* sp. nov. is suspected to share the same tolerance, there was not yet an experiment designed to test this ability in this clade.

## Supporting information

S1 Fig Sonograms of audio files: S1–S10 Audios. (PDF)

S1 Audio *Buergeria japonica*, NMNS 19899, long calls (Type 1A). (WAV)

S2 Audio *Buergeria japonica*, NMNS 19907, long calls (Type 1A) + short calls. (WAV)

S3 Audio *Buergeria otai*, holotype, NMNS 19819, long calls (Type 1B) + short calls + long calls (Type 2). (WAV)

S4 Audio *Buergeria otai*, paratype, NMNS 19824, long calls (Type 1B) + short calls. (WAV)

S5 Audio *Buergeria otai*, paratype, NMNS 19815, long calls (Type 1B) + short calls. (WAV)

S6 Audio *Buergeria japonica*, NMNS 19806, long calls (Type 1B) + short calls + long calls (Type 2). (WAV)

S7 Audio *Buergeria japonica*, NMNS 19808, short calls + long calls (Type 2). (WAV)

S8 Audio *Buergeria otai*, holotype, NMNS 19812, long calls (Type 2). (WAV)

S9 Audio *Buergeria otai*, paratype, NMNS 19817, short calls + long calls (Types 2). (WAV)

S10 Audio *Buergeria otai*, paratype, NMNS 19822, long calls (Type 2) + short calls. (WAV)
