# Correction: Acoustic differentiation and behavioral response reveals cryptic species within *Buergeria* treefrogs (Anura, Rhacophoridae) from Taiwan

**DOI:** 10.1371/journal.pone.0193040

**Published:** 2018-02-13

**Authors:** Ying-Han Wang, Yu-Wei Hsiao, Ko-Huan Lee, Hui-Yun Tseng, Yen-Po Lin, Shohei Komaki, Si-Min Lin

In the Methods section, the Nomenclatural Acts sub-section is missing. The correct sub-section is:

## Nomenclatural Acts

The electronic edition of this article conforms to the requirements of the amended International Code of Zoological Nomenclature, and hence the new names contained herein are available under that Code from the electronic edition of this article. This published work and the nomenclatural acts it contains have been registered in ZooBank, the online registration system for the ICZN. The ZooBank LSIDs (Life Science Identifiers) can be resolved and the associated information viewed through any standard web browser by appending the LSID to the prefix “http://zoobank.org/”. The LSID for this publication is: urn:lsid:zoobank.org:pub: 092F06B7-B0F4-40F2-BF6A-4077A590766D. The electronic edition of this work was published in a journal with an ISSN, and has been archived and is available from the following digital repositories: PubMed Central, LOCKSS.

In the Species description sub-section of the Discussion section, the LSID for *Buergeria otai* sp. nov. is missing. The correct LSID is: urn:lsid:zoobank.org:act:BAF9221D-D0BE-4441-AC2B-46BFF9057E7D.

## References

[1] Wang Y-H, Hsiao Y-W, Lee K-H, Tseng H-Y, Lin Y-P, Komaki S, et al (2017) Acoustic differentiation and behavioral response reveals cryptic species within *Buergeria* treefrogs (Anura, Rhacophoridae) from Taiwan. PLoS ONE 12(9): e0184005 https://doi.org/10.1371/journal.pone.0184005 28877201 10.1371/journal.pone.0184005 PMC5587266

