# Supplementary material for: Acoustic differentiation and behavioral response reveals cryptic species within Buergeria treefrogs (Anura, Rhacophoridae) from Taiwan
Source: PLoS One. 2017 Sep 6;12(9):e0184005. doi: 10.1371/journal.pone.0184005 (PMC5587266; doi:10.1371/journal.pone.0184005)
Supplement: S1 Fig — (PDF) [file pone.0184005.s001.pdf]

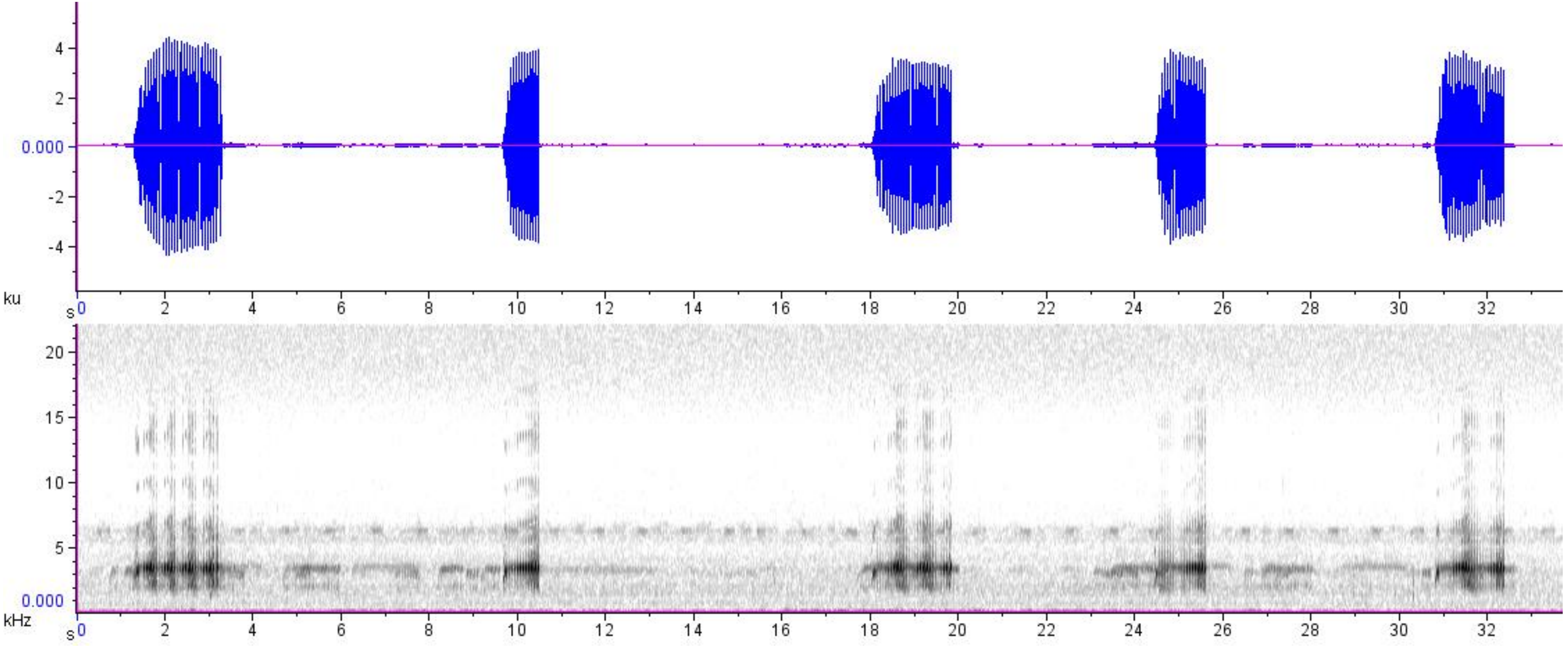

S1 Audio: *Buergeria japonica*, NMNS 19899
Locality: ZhouShui Stream, northwestern Taiwan
Call type: Long calls (Type 1A)

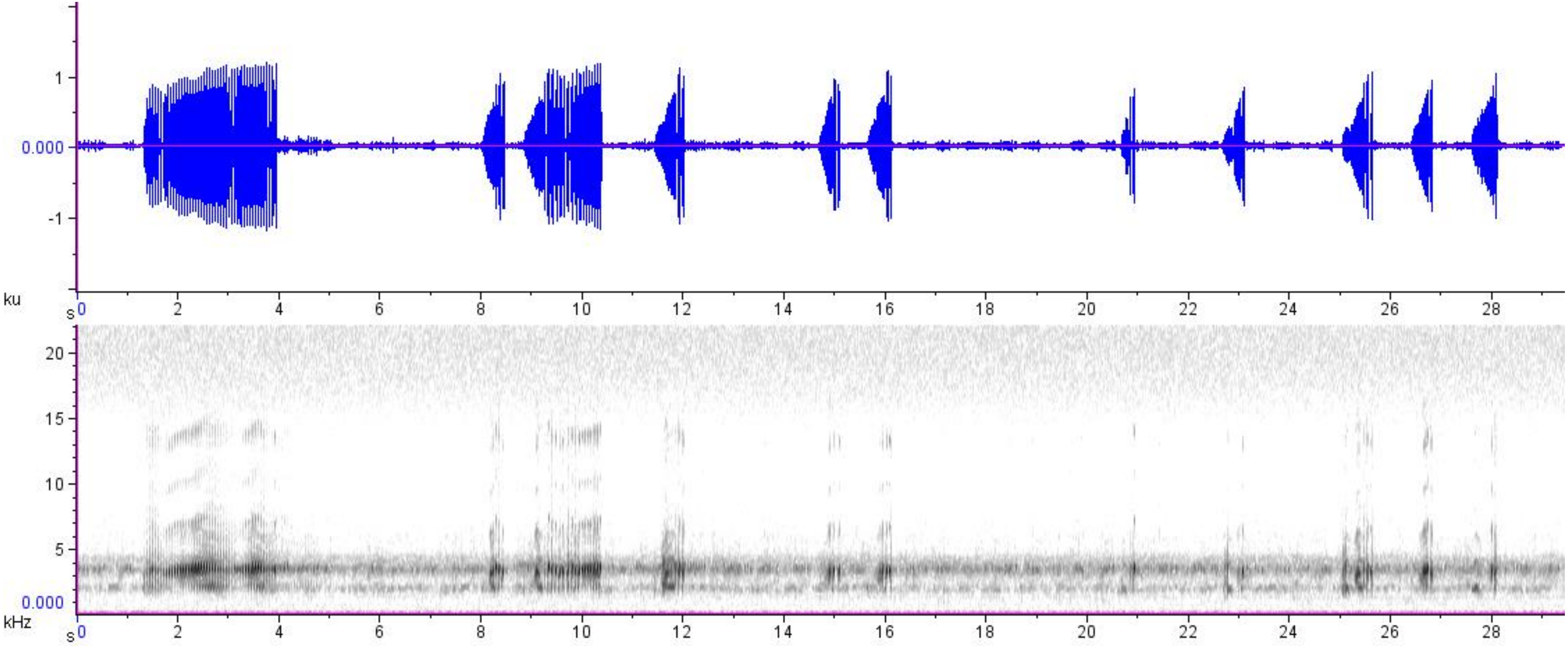

S2 Audio: *Buergeria japonica*, NMNS 19907
Locality: ZhongGang Stream, northwestern Taiwan
Call type: Long calls (Type 1A) + short calls

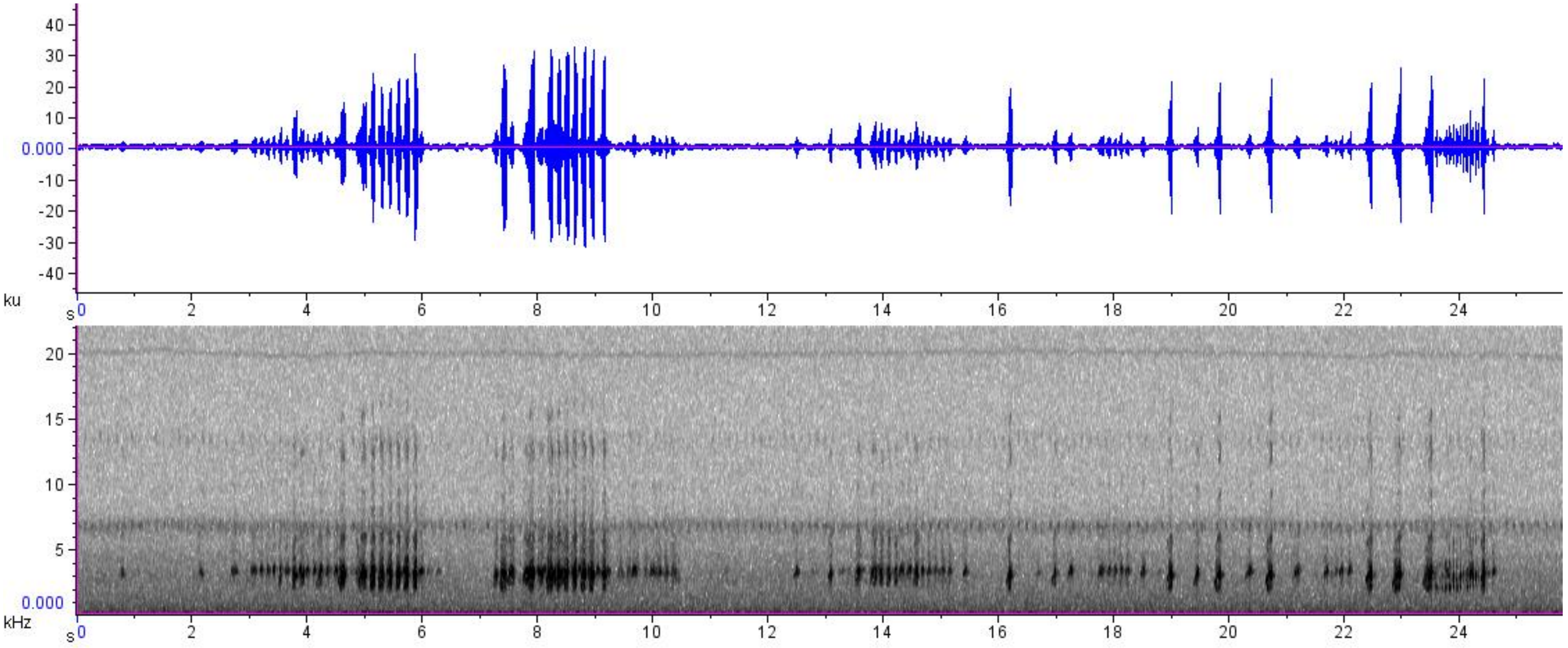

S3 Audio: *Buergeria otai*, holotype, NMNS 19819
Locality: DongGang Stream, southern Taiwan
Call type: Long calls (Type 1B) + short calls + long calls (Type 2)

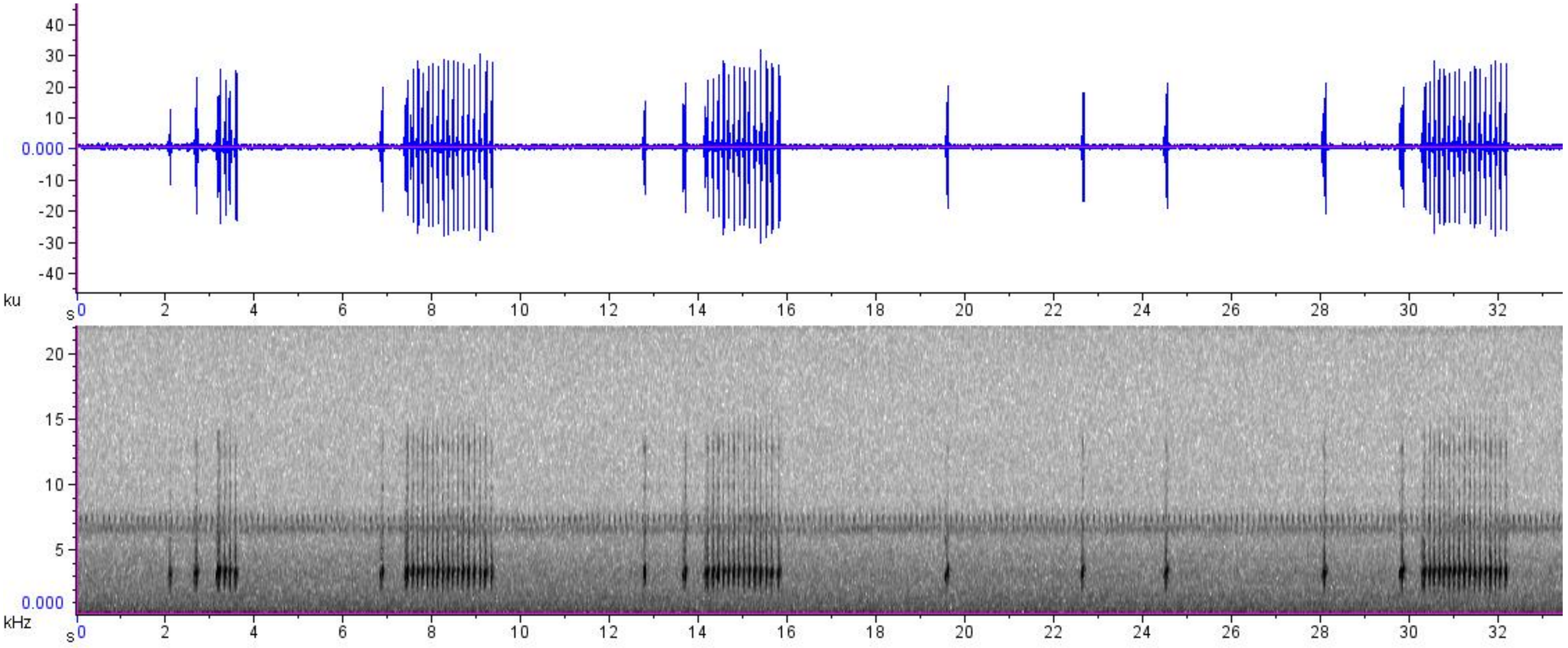

S4 Audio: *Buergeria otai*, paratype, NMNS 19824
Locality: DongGang Stream, southern Taiwan
Call type: Long calls (Type 1B) + short calls

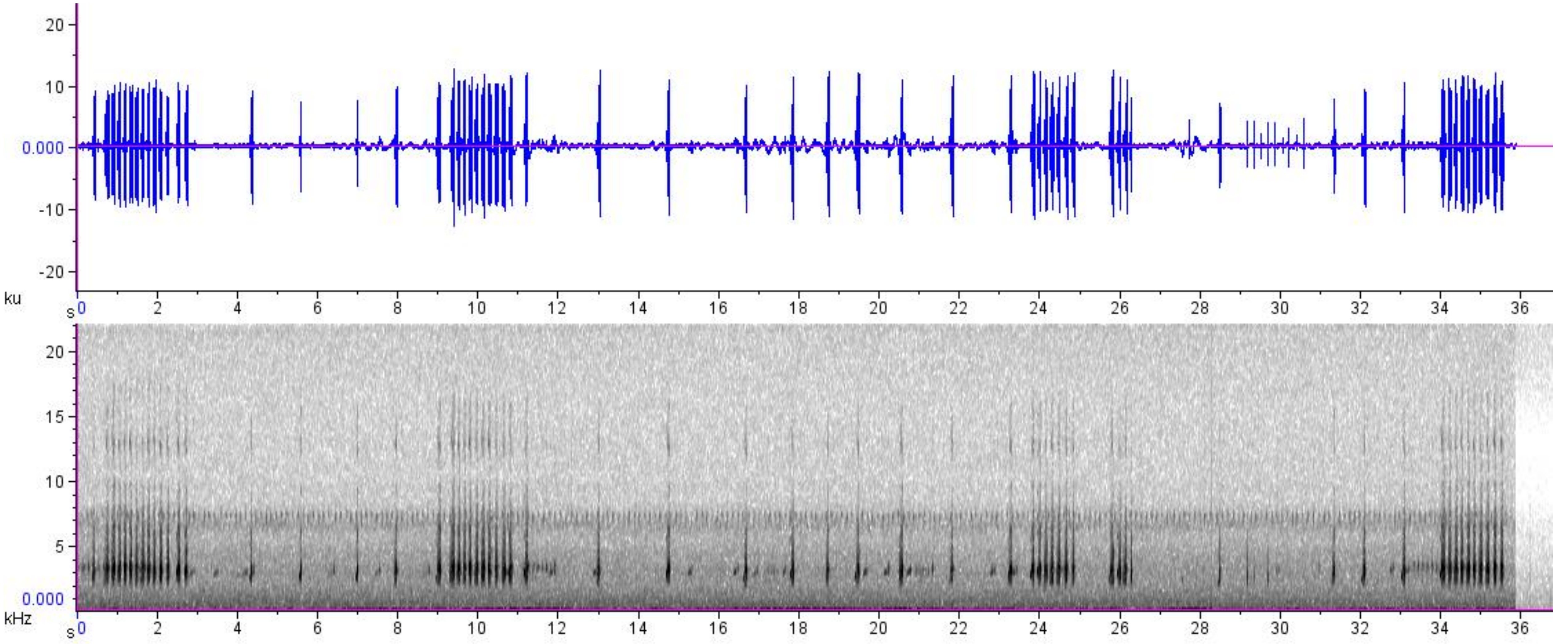

S5 Audio: *Buergeria otai*, paratype, NMNS 19815
Locality: DongGang Stream, southern Taiwan
Call type: Long calls (Type 1B) + short calls

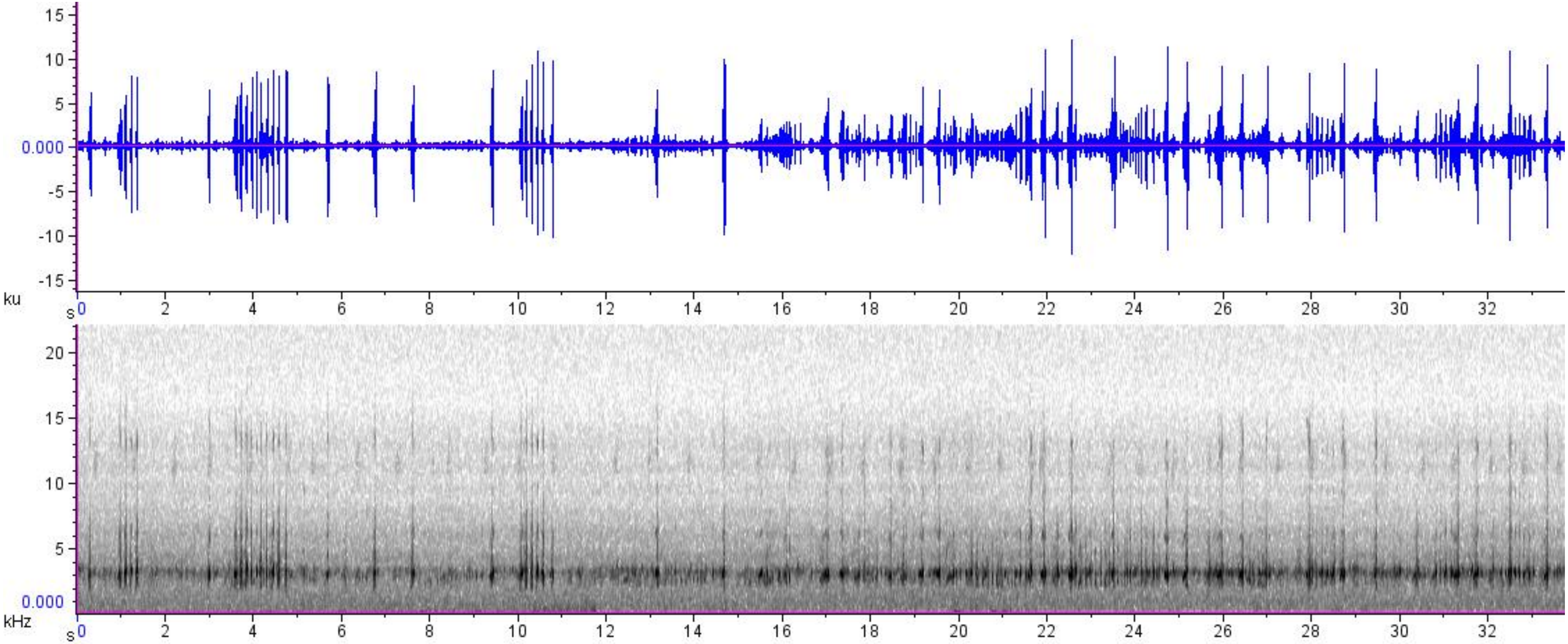

S6 Audio: *Buergeria otai*, paratype, NMNS 19806
Locality: MeiLun Stream, eastern Taiwan
Call type: Long calls (Type 1B) + short calls + long calls (Type 2)

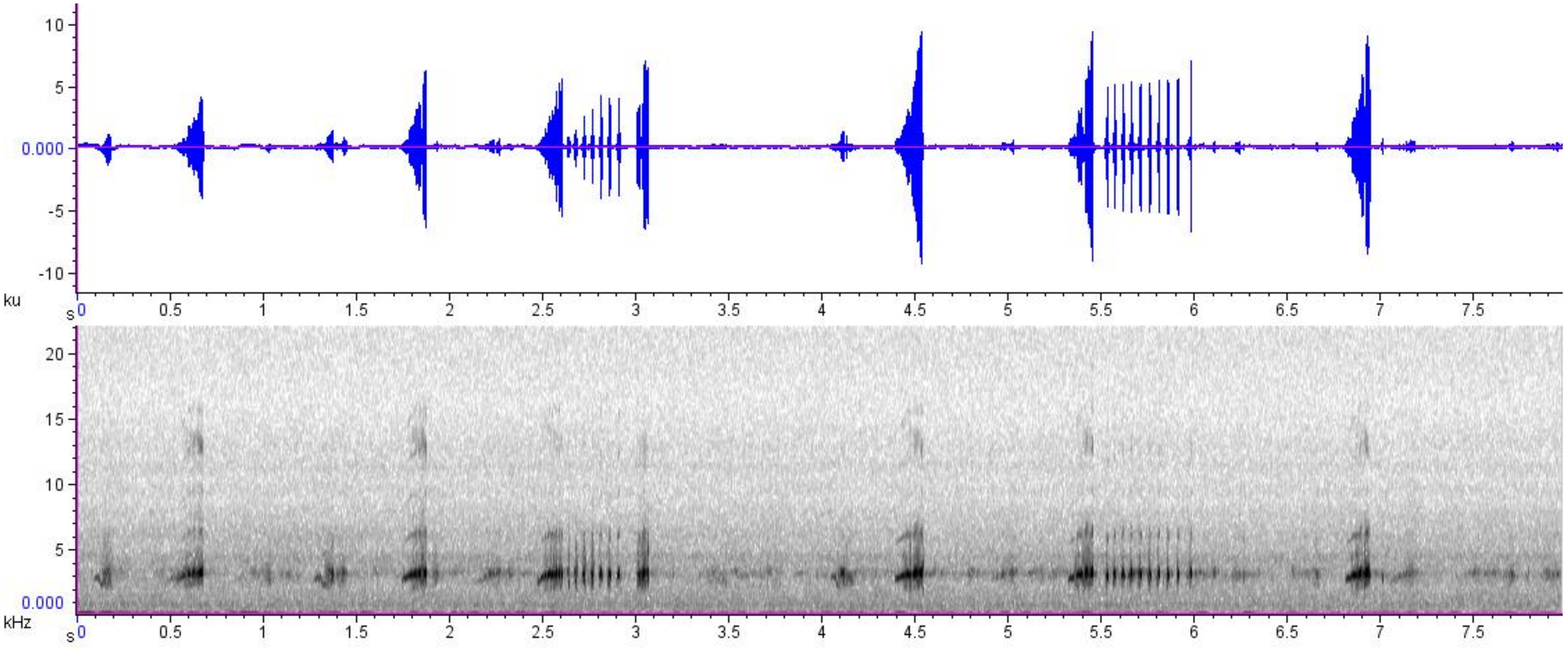

S7 Audio: *Buergeria otai*, paratype, NMNS 19808
Locality: MeiLun Stream, eastern Taiwan
Call type: Short calls + long calls (Type 2)

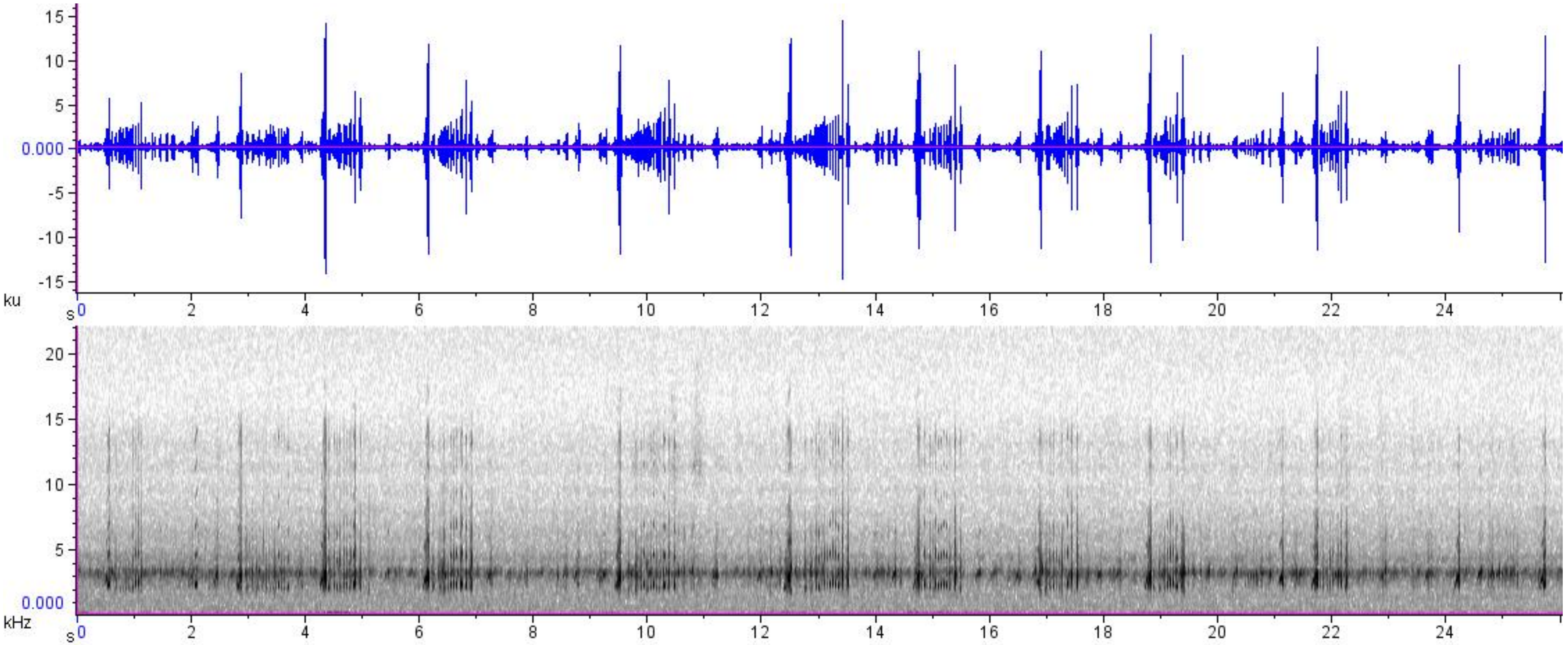

S8 Audio: *Buergeria otai*, paratype, NMNS 19812
Locality: NanAo Stream, eastern Taiwan
Call type: Long calls (Type 2)

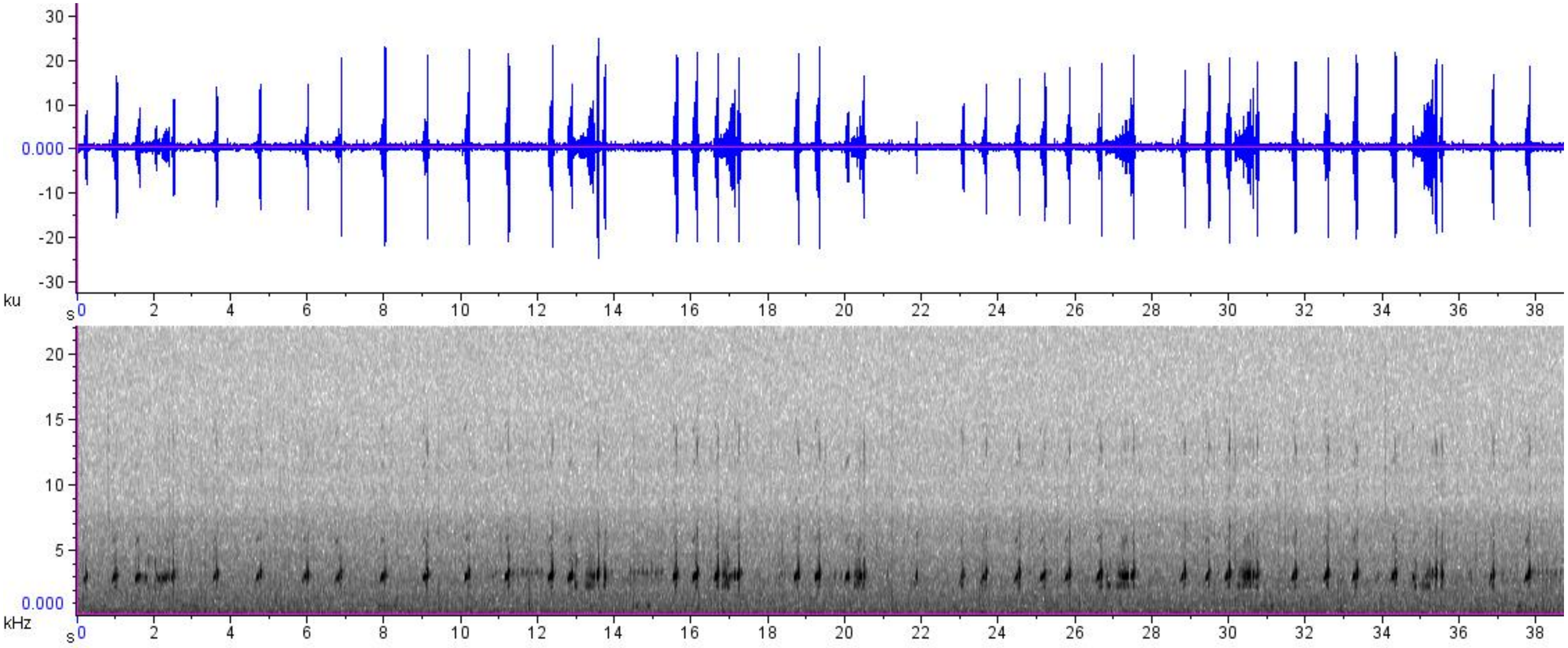

S9 Audio: *Buergeria otai*, paratype, NMNS 19817
Locality: DongGang Stream, southern Taiwan
Call type: Short calls + long calls (Types 2)

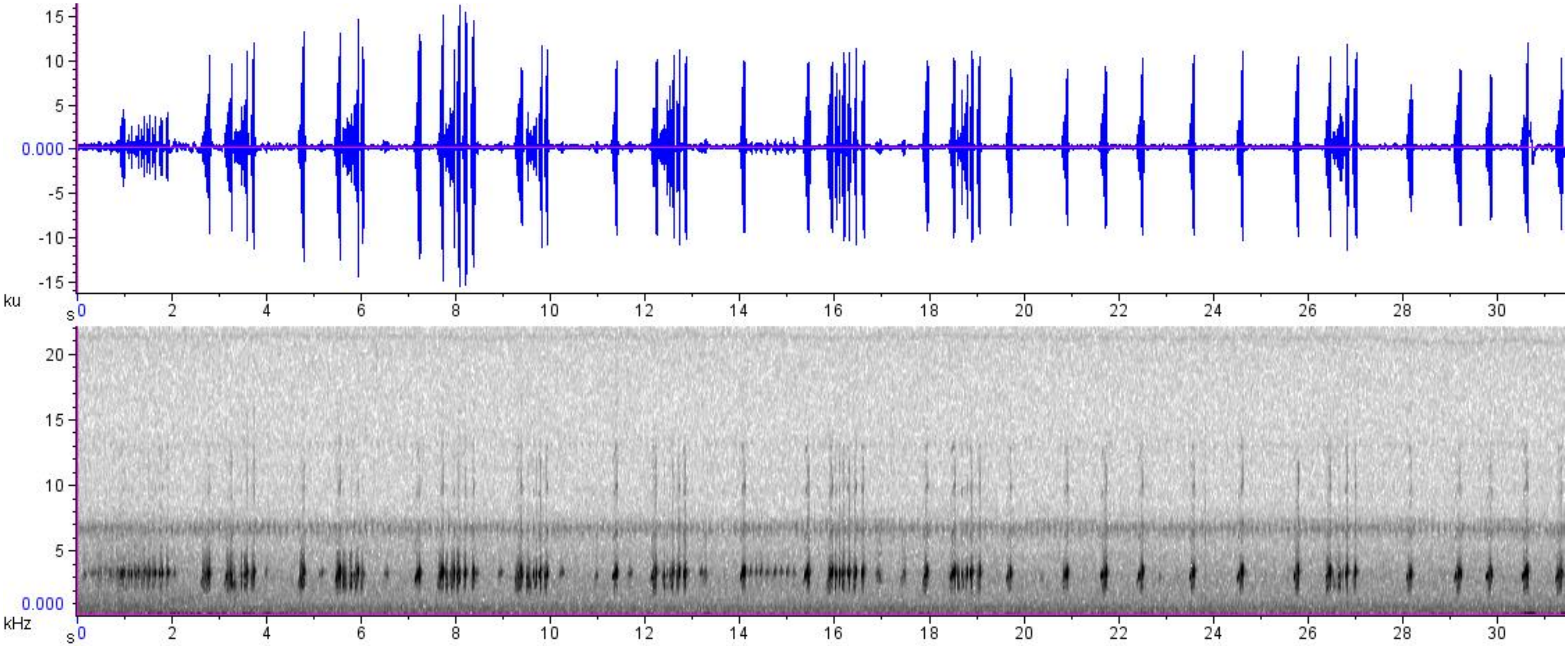

S10 Audio: *Buergeria otai*, paratype, NMNS 19822
Locality: DongGang Stream, southern Taiwan
Call type: Long calls (Type 2) + short calls
